# Comparison of Combined Oral Contraceptives and a Transdermal Estrogen Patch With Oral Progesterone: Treatment of Abnormal Uterine Bleeding in Adolescents

**DOI:** 10.7759/cureus.72218

**Published:** 2024-10-23

**Authors:** Hasan Bora Ulukapi, Enver Simsek

**Affiliations:** 1 Department of Pediatrics, Eskisehir Osmangazi University Faculty of Medicine, Eskisehir, TUR; 2 Department of Pediatric Endocrinology, Eskisehir Osmangazi University Faculty of Medicine, Eskisehir, TUR

**Keywords:** abnormal uterine bleeding, adolescent, combined oral contraceptive, ovulatory dysfunction, progestin, transdermal estrogen patch

## Abstract

Objective

Abnormal uterine bleeding (AUB) is a frequent complaint in adolescents. Ovulatory dysfunction (AUB-O) is the most common etiology of AUB. We aimed to compare possible treatment modalities for idiopathic AUB-O, the most common reason for AUB-O in adolescents.

Methods

Thirty patients who were treated with either 30 μg ethinyl estradiol/3 mg drospirenone combined oral contraceptive (group A) or 1.95 mg 17β-estradiol transdermal patch and 5 mg oral medroxyprogesterone acetate combined treatment (group B) for six months in the pediatric endocrinology department between years 2017 and 2019 were enrolled in our study retrospectively, and a questionnaire was performed on them to assess their treatment satisfaction.

Results

In the first three months of treatment, four (26.6%) patients reported intermenstrual bleeding (IMB), three (20%) reported abdominal pain, two (13.3%) reported nausea-vomiting, and one (6.7%) reported headaches in group A, while eight (53.3%) patients reported IMB, six (40%) reported abdominal pain, two (13.3%) reported nausea-vomiting, and two (13.3%) reported headaches in group B. No major side effects were reported to cause cessation of treatment in any treatment group, but fewer mild side effects were reported in group A. Group A had better treatment compliance and satisfaction. Menstrual irregularity stopped after two months of treatment in both groups, but one (6.6%) and three (20.0%) patients reported that AUB repeated within three months of treatment cessation in group A and group B, respectively.

Conclusion

Immaturity of the hypothalamo-pituitary-ovarian axis in adolescence can present with a wide range of symptoms and proves challenging to choose the proper treatment regimen.

## Introduction

In adolescents, any deviation in hypothalamo-pituitary-gonadal axis maturation may result in abnormal uterine bleeding (AUB) [1]. While anovulatory cycles in adolescents right after menarche are common, 90% of cycles stay within normal limits. However, heavy menstrual bleeding is more frequent in adolescents (37%) compared to adults [2,3].

The classification of AUBs was redefined by the International Federation of Obstetrics and Gynecology (FIGO) in 2011 with the acronym PALM-COEIN. The letter ‘O’ in COEIN defines AUB due to ovulatory dysfunction (AUB-O). While usually no etiology can be shown in adolescent patients with AUB-O, endocrinopathies such as polycystic ovary syndrome, hypothyroidism, hyperprolactinemia, obesity, anorexia, and late-onset congenital adrenal hyperplasia are also common etiologies [4].

Treatment of AUB-O typically involves inducing endometrial proliferation with estrogens and stabilizing the endometrium with progestins, aiming to stop heavy bleeding and prevent recurrence while discovering the etiology. With this main understanding, treatment approaches are highly variable, including combined estrogen and progestin oral contraceptives (COC), progestin-only methods, and estrogen-only methods (transdermal patch) [5,6]. While research on contraceptive efficacy (Pearl index) is extensive, studies focusing on AUB treatment effectiveness in adolescents are limited. Finally, rather than an objective assessment of the amount of bleeding such as pictorial blood loss assessment charts, subjective assessments of patient happiness and quality of life have become a more important factor in choosing treatment [7].

This study compares two treatments for AUB due to idiopathic AUB-O in adolescents: combined oral contraceptives and a regimen combining a transdermal estrogen patch with oral progesterone.

## Materials and methods

Cases

**Figure 1:**
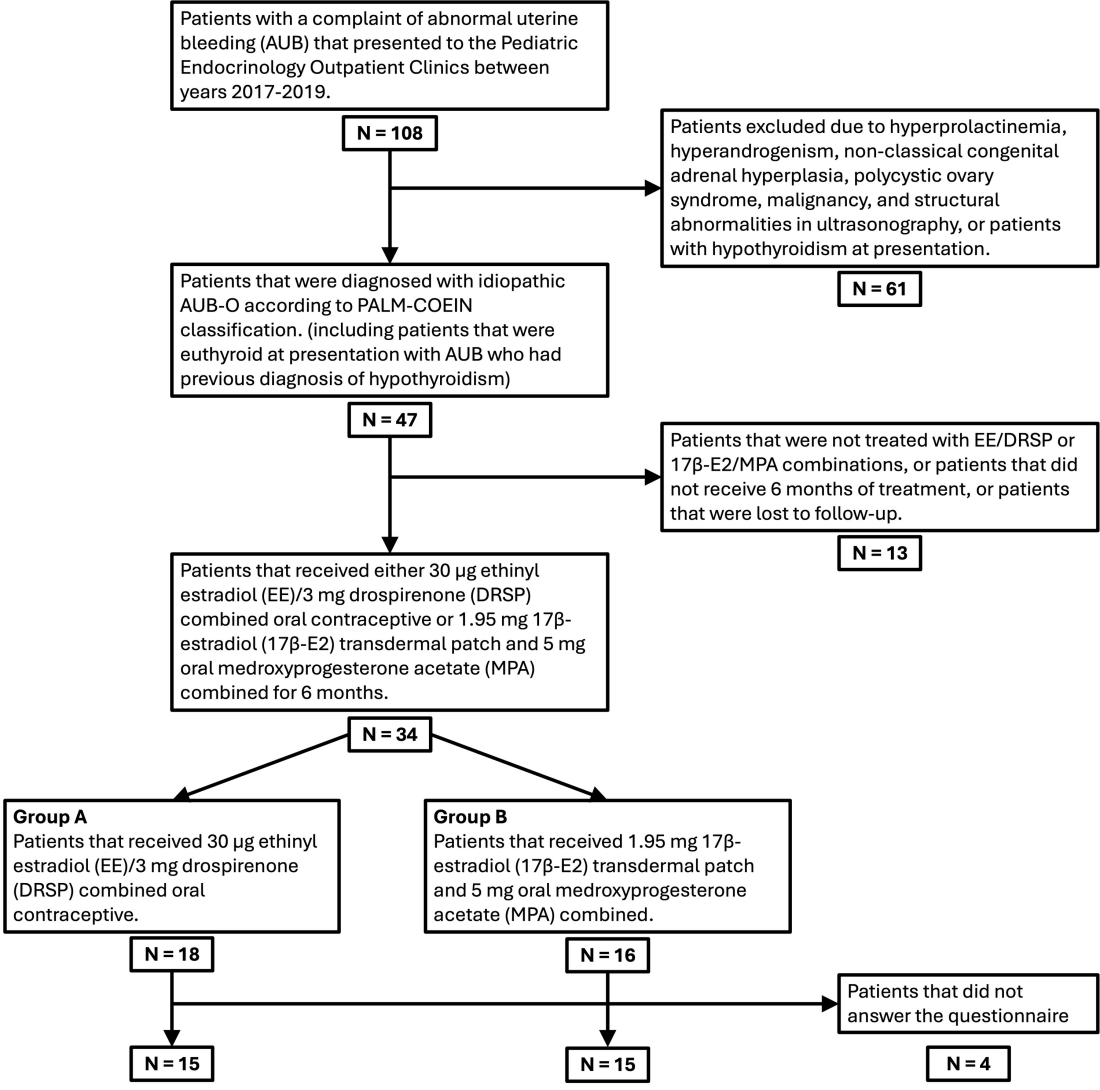
Patient selection, inclusion, and exclusion using medical records

A cohort of 30 adolescents who were followed up for six months in the Eskisehir Osmangazi University Pediatric Endocrinology Department, Turkey, between the years 2017 and 2019 with the complaint of AUB and diagnosed with idiopathic AUB-O were retrospectively included in the study. Two treatment groups, each with 15 patients, were formed that received either 30 μg ethinyl estradiol (EE)/3 mg drospirenone (DRSP) combined oral contraceptive or 1.95 mg 17β-estradiol (17β-E2) transdermal patch and 5 mg oral medroxyprogesterone acetate (MPA) combined (Figure 1).

The sample size was based on power analysis from similar AUB studies assuming a large effect size, estimating 10-13 patients per group for a power of 80-90%. With 15 patients per group, the study was considered adequately powered.

The patient’s diagnoses were confirmed by patient records of laboratory testing and imaging techniques at presentation. These tests included prothrombin time, international normalized ratio, activated partial thromboplastin time, follicular phase levels of follicle-stimulating hormone (FSH), luteinizing hormone (LH), estradiol (E2), prolactin, beta-human chorionic gonadotropin (βHCG), 17-hydroxy progesterone (17-OH-P), dehydroepiandrosterone sulfate (DHEA-S), total testosterone, thyroid stimulating hormone (TSH), and free thyroxine (fT4) in addition to obstetric ultrasound imaging. For patients with unpredictably irregular menstruation, the follicular phase levels could not be determined. Patients with a diagnosis of hypothyroidism who were euthyroid under treatment of levothyroxine and presented with AUB were included in the study.

Exclusion criteria encompassed hyperprolactinemia, hyperandrogenism, non-classical congenital adrenal hyperplasia, polycystic ovary syndrome, malignancy, and structural abnormalities in ultrasonography. Anemia status at presentation was noted, and patients were classified based on their post-discharge treatment regimens. Hemoglobin levels had been monitored at the third and sixth-month marks.

Presentation complaints were classified into prolonged menstruation (>8 days of cycles), HMB (>80 ml of bleeding per cycle), frequent menstrual bleeding (<21 days between cycles), and infrequent menstrual bleeding (>45 days between cycles) [8].

**Figure 2:**
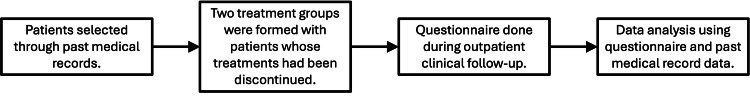
Study design

Treatment adherence was assessed, including any initial non-compliance rectified within the study period. A questionnaire on these past treatments was done during selected patients' outpatient clinical follow-ups and evaluated treatment adherence challenges, side effects (headaches, abdominal pain, nausea-vomiting, intermenstrual bleeding (IMB)), and the duration to regular menses or cessation of HMB. Recurrence of symptoms post-treatment cessation within six months was also examined (Figure 2) (Supplementary material 1).

The study protocol was in line with the Declaration of Helsinki and was approved by the Non-Interventional Clinical Research Ethics Committee of our university on June 25, 2019, with approval number 12.

Statistical analysis

Data analysis was done using IBM SPSS Statistics for Windows, Version 21 (Released 2012; IBM Corp., Armonk, New York, United States). Data on qualitative variables were shown as frequency and percentages, while data on quantitative variables that had a normal distribution were shown as mean ± standard deviation. The distribution of quantitative variables was assessed with the Shapiro-Wilk test. A comparison of two groups that had normal distribution was done using the t-test, and two groups that did not have normal distribution were compared using the Mann-Whitney U test. Relationships between qualitative variables were evaluated using chi-square analysis. A comparison of repeated measurements of qualitative variables was done using the McNemar test. P-values less than 0.05 were considered statistically significant.

## Results

Thirty adolescents presenting with AUB, categorized into AUB-O, were included in the study. Two treatment groups, each with 15 patients, received either 30 μg EE/3 mg DRSP combined oral contraceptive (group A) or 1.95 mg 17β-E2 transdermal patch combined with 5 mg oral MPA (group B).

Baseline characteristics, including age, BMI, anemia prevalence, and hospitalization at presentation, showed no significant differences between the groups (Table 1) (p > 0.05). Four (13.33%) patients had a diagnosis of hypothyroidism, but they were euthyroid under levothyroxine treatment at the time of presentation with AUB. One patient was diagnosed with autism and another with familial Mediterranean fever.

**Table 1:** General data of patients at presentation BMI: body mass index; SD: standard deviation

Patient characteristics	Group A	Group B	p-value
Patients, n	15	15	
Age (year), mean ± SD	14.03 ± 1.79	14.65 ± 1.21	0.28
BMI percentile, mean ± SD	52.76 ± 31.99	56.65 ± 33.38	0.75
Patients presented with anemia, n (%)	7 (46.7)	5 (33.3)	0.71
Patients hospitalized at presentation, n (%)	3 (20.0)	2 (13.3)	1.00

No statistically significant difference between the groups was found regarding chief complaints (p > 0.05). The most common complaint was a longer duration of menstruation in both groups, with a total of 23 patients (76.6%).

When patients were evaluated according to their hemoglobin (Hb) levels at presentation, eight (26.7%) had severe (Hb < 10 g/dL), six (20.0%) had moderate (Hb 10-12 g/dL), and 16 (53.3%) had mild HMB (Hb ≥ 12 g/dL). Five of the eight patients who presented with severe HMB were hospitalized.

No statistical differences were found between LH, FSH, E2, 17-OH-P, prolactin, DHEA-S, or total testosterone levels of the two groups (p > 0.05).

Normal menstrual cycles were restored within three months of treatment in both groups. While there was no statistical difference, more patients' (14 patients, 93.3%) menstrual cycles were normal after the first month of treatment in group A, compared to group B (11 patients, 73.3%) (p = 0.34). Menstrual cycles of the patients in group A normalized significantly more (13 patients, 86.7%) after just one cycle compared to group B (7 patients, 46.7%) (p = 0.02). Similarly, while there was no statistical significance, menstrual cycles were restored faster in group A compared to group B in general (p = 0.07) (Table 2). Patients in both groups reported a significant decrease in the amount of menstrual bleeding within two months of treatment (Table 3).

**Table 2:** Time to restore normal menstrual cycles in each treatment group

Patients	Group A n (%)	Group B n (%)	p-value
Patients with restored normal cycles after first cycle (n)	13 (86.7)	7 (46.7)	0.07
Patients with restored normal cycles after second cycle (n)	2 (13.3)	4 (26.7)	
Patients with restored normal cycles after third cycle (n)	0 (0.0)	3 (20.0)	
Patients with restored normal cycles after fourth cycle (n)	0 (0.0)	0 (0.0)	
Patients with restored normal cycles after fifth cycle (n)	0 (0.0)	1 (6.7)	

**Table 3:** Time to decreased menstrual bleeding

Patients	Group A n (%)	Group B n (%)	p-value
Patients with decreased menstrual bleeding within first month (n)	10 (66.6)	8 (53.3)	0.71
Patients with decreased menstrual bleeding within second month (n)	5 (33.3)	7 (46.7)	

**Figure 3:**
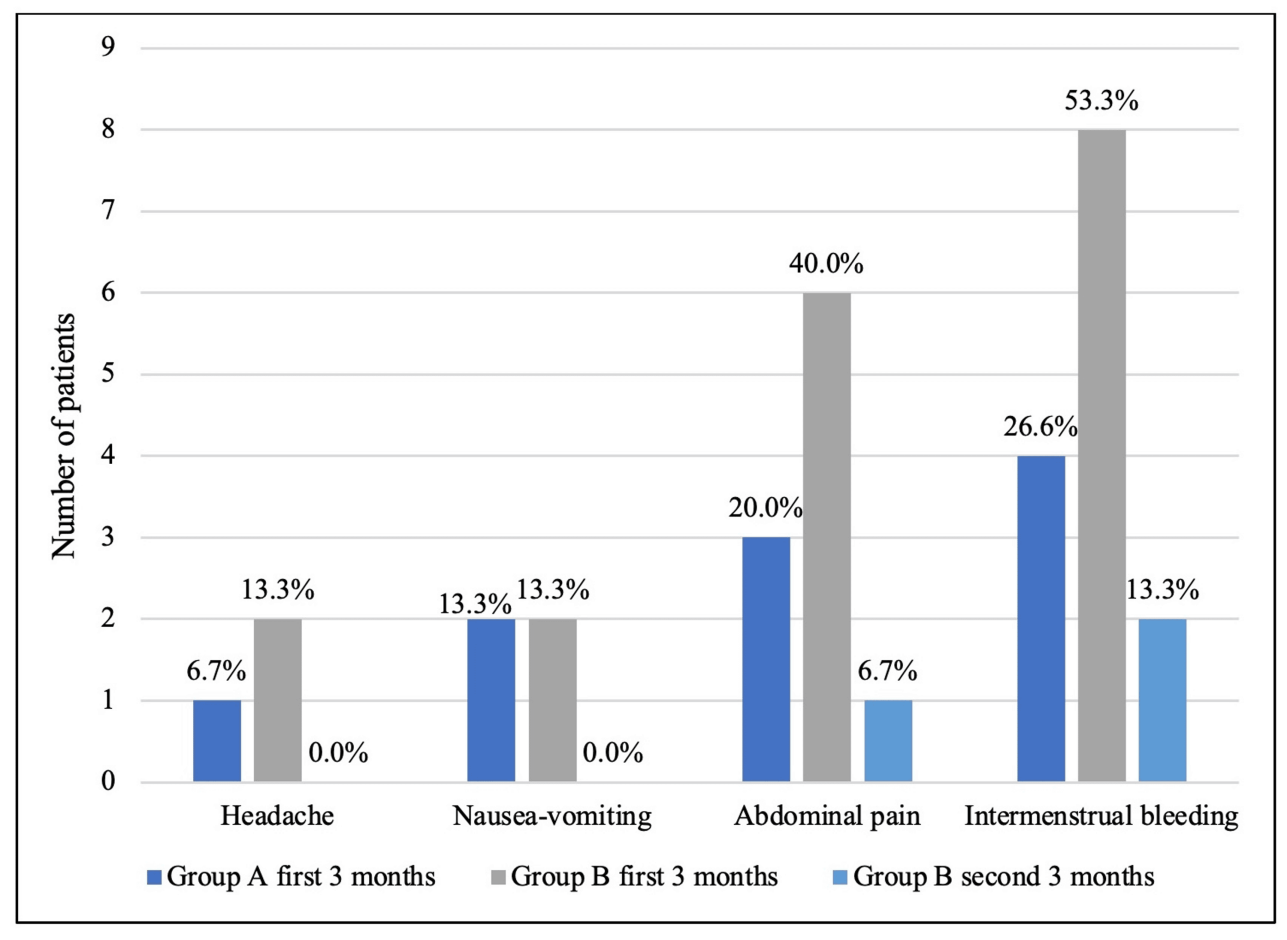
Reported side effects in first three months and second three months of treatment

A total of 19 (63.3%) patients reported side effects, none of which were life-threatening. No patients developed severe side effects such as hypertension, venous thromboembolism, or stroke. While there was no statistically significant difference, a smaller number of mild side effects were reported in group A (8 patients, 53.3%) compared to group B (11 patients, 73.3%) (p = 0.45). While 19 patients reported side effects in the first three months of treatment, only three patients reported side effects in the second three months of treatment (Figure 3).

Within the first three months of treatment, abdominal pain was more prevalent in group B with six patients (40.0%), while it was reported by three patients (20.0%) in group A, though this was not statistically significant (p > 0.05). No side effects were reported in the second three months from group A.

Two out of eight patients (25.0%) in group B that reported IMB under treatment had this complaint for the whole six months of treatment; the patients that did not have this side effect did not develop it in the second three months of the treatment either. The number of patients who reported IMB in the second three-month period of treatment was higher among those who also reported such bleeding in the first three months (p = 0.03). This was similar to the patients who reported abdominal pain in group B (p = 0.03).

No correlation was found between laboratory values and side effects or symptom recurrence post-treatment (p > 0.05).

While no statistical significance was found between presentation complaints and treatment side effects, patients who presented with either a longer duration of menstruation or HMB reported having more IMB side effects within the first three months of treatment (p = 0.19 and p = 0.12, respectively). Patients who presented with HMB reported more headaches, though this was not statistically significant (p = 0.28).

When the increase in hemoglobin levels in patients who presented with anemia was compared, group A showed a 5.85 ± 3.01 g/dL increase, while group B showed 4.52 ± 3.19 g/dL. Both groups showed a marked increase in hemoglobin levels post-treatment, with no significant difference between them (p = 0.54).

Four patients reported recurrence of complaints within three months after treatment cessation. While there was no statistically significant difference between the two groups, group B reported more (3 patients, 20.0%) recurrences than group A (1 patient, 6.6%) (p = 0.59).

While 14 (93.3%) patients reported that they were satisfied with the treatment in group A, this number was 10 (66.7%) in group B (p = 0.17).

## Discussion

AUB is a frequent complaint in adolescents [9]. It is reported that menstrual problem prevalence in adolescents is up to 73% and HMB prevalence is 12-37% [2,10,11]. AUB presents half of the adolescent gynecological problems and affects 14-25% of fertile women [12,13]. It was shown that AUB-O patients form 57% of the patients with AUB, and 5% of these patients are adolescents [14]. Menstrual history is accepted as a vital sign and suggested that it be assessed in every examination [7].

Lately, treatment guidelines have expressed the importance of different treatments for various etiologies of AUB [8,13,15]. While AUB-O presents a major etiology of AUB in all ages as well as adolescents, immaturity of the hypothalamo-pituitary-ovarian axis is an especially important etiology in this age group [15].

Recent approaches in assessing AUB treatment success extend beyond just measuring bleeding reduction to include subjective parameters [7]. These include abdominal pain, predictability of menstruation, impact on daily activities, and even clothing choices influenced by menstrual concerns [16]. Our study embraced this broader evaluation framework, incorporating assessments of abdominal pain, nausea, vomiting, headaches, IMB, and symptom recurrence post-treatment cessation.

Similar to a study by Elmaoğulları et al. from Turkey, evaluating 22 adolescents, where the age at presentation was 13.9 ± 1.7, our patients’ presentation age was 14.3 ± 1.5 [11]. Though they reported that the number of adolescents presented with HMB was 14 (63.7%), this percentage in our study was lower to 26.7% with eight patients. About 19 (86%) patients were reported to receive 30 μg EE-desogestrel monophasic COC treatment in this study, and this treatment had a 100% success rate (22 patients); similarly, our study, which included the EE-DRSP COC treatment group, had a 93.4% success rate (14 patients).

A study comparing norethisterone (NET) and a COC that includes 50 μg EE on pubertal menorrhagia by Patel et al. reported recurrence of symptoms in three (11%) patients three months after cessation of NET treatment and a higher 32% (9 patients) recurrence after COC treatment [17]. In the COC group, six (22%) patients reported nausea-vomiting, three (8%) reported IMB, and abdominal pain was not reported. In our study, with a 26.6% frequency (4 patients), IMB was the most common complaint in the first three months of treatment, while nausea-vomiting was reported by two (13.3%) patients and less than what Patel et al. reported. While our study showed no difference in hemoglobin increase in the two treatment groups, the study by Patel et al. reported a higher hemoglobin increase in the NET group compared to COC.

Creatsas et al. compared tenoxicam versus high-dose 50 μg EE-linestrenole COC for the treatment of AUB-O in 48 adolescents and evaluated parameters up to cessation of acute menorrhagia [18]. Eighteen (72%) patients who received COC treatment were reported to have severe gastrointestinal symptoms and vertigo. In our study, we used a daily single dose of COC and reported a lower 33.3% (5 patients) of mild gastrointestinal symptoms. Creatsas et al. defined treatment failure as a necessity for transfusions or curettage and reported 10 (40%) patients needing such interventions in the COC treatment group. In contrast, treatment failure was defined as symptom recurrence within three months post-treatment, and this was reported to be one patient (6.6%) in group A and three (20%) in group B, which were much lower than what Creatsas et al. reported. This difference could be attributed to different treatment aims. Our study did not evaluate this acute period of HMB in our participants and compared the following treatments after the patient was stabilized.

A study by Sen et al., including 32 adolescents with AUB-O together with adults, compared a COC consisting of 30 μg EE-LNG with NET treatments [19]. The most common side effect was reported to be nausea-vomiting in 12 (24%) patients, while headaches were seen in three (6%) patients and IMB was seen in two (4%) patients. IMB was much higher in our study with four (26.6%) patients. The difference in the progestin portion of COC, LNG versus DRSP, and the inclusion of adult patients may account for this difference.

A study by Jain et al. comparing EE-etonorgestrel containing vaginal ring versus 30 μg EE-LNG containing COC for adolescent and adult patients reported only one (3%) patient with IMB in patients using vaginal ring and nine (30.3%) patients with abdominal pain [20]. COC treatment in the same study was reported to have four (14%) patients with IMB, headaches, and abdominal pain. Our study reported more frequent IMB with four (26.6%) and eight (53.3%) patients in each group, and abdominal pain with three (20%) and six (40%) patients in each group.

In a study by Abu Hashim et al. on HMB in young adults comparing EE-etonorgestrel containing vaginal ring and NET treatments, two (4.2%) patients reported IMB that was treated with vaginal ring and six (12.8%) patients using NET [21]. Both values were lower than our reported IMB frequencies.

Munro et al. conducted a study on acute HMB in adults, comparing the use of a COC containing 35 μg EE-NET and MPA treatments [22]. They reported four (24%) patients with recurrence of symptoms after COC treatment and two (12%) patients with recurrence after MPA treatment. These findings align with the recurrence rates observed in our study.

No study was found that included the same treatment used in group B, though 17β-E2 treatment was assessed using a higher dose in premenopausal women in the study by Tranquilli et al. [23]. Holtorf’s study reported fewer side effects in 17β-E2 treatment compared to its synthetic derivatives, while MPA was reported to have more side effects [24]. Treatment dosage and patient age largely differ from our study and can be the cause for the difference in the side effect profile. It is quite apparent that these parameters should be considered in treatment choice.

The frequency of IMB in our study was much higher than in other studies. Each group had four (26.6%) and eight (53.3%) patients reporting IMB, while other studies reported between 3% and 22%. This difference could be attributed to recall bias or differences in included age groups. Abdominal pain and nausea-vomiting were reported similar to previous studies as 3-6 (20-40%) and 2 (13.3%) patients, respectively. Headache reports were also similar to previous literature as one (6.7%) and two (13.3%) patients in each group [12,17-21].

Our study's unique contribution lies in its focus on idiopathic AUB-O in adolescents, a relatively underrepresented group in AUB research. The six-month follow-up period provides a more extended observation window than many studies, though longer-term follow-ups are necessary for a complete understanding of treatment efficacy and safety [17,20,21,25,26]. Most studies on AUB were on COCs or vaginal contraceptives, while no studies on transdermal estrogen and oral progesterone combined treatment were found.

In assessing treatment success, the duration of cessation of acute menorrhagia was a common endpoint [18]. Our study included this parameter as well as the duration of normalization of menstruation order as a new treatment success parameter. Both treatment groups were evaluated as successful in both of these parameters in our study within two months. Another commonly used parameter, patient satisfaction, was also evaluated, and differing from studies on adults, with a 93.3% satisfaction rate (14 patients), COC treatment was highly satisfactory [12,17,22,25].

The variation in side effects reported across different studies highlights the complex interplay of factors influencing treatment response, including the type of estrogen or progesterone, administration route, and individual patient characteristics. The fact that studies on adolescents did not report on the side effect frequencies in detail and studies that included all ages did not report side effect frequencies on different age groups complicates comparing data [18-20,27]. There were no studies in the literature reporting on side effects in different time periods in adolescents. In addition, in general, as the Cochrane meta-analyses have reported, there is a lack of randomized controlled trials with a larger population on the treatment of AUB [28-30].

While COC treatment demonstrated fewer side effects, none of the treatment groups reported any side effects severe enough that would necessitate treatment cessation. Both combined contraceptive treatments achieved normal menstrual cycles within two months of treatment. Although COC treatment proved more effective in normalizing cycles after the first cycle, transdermal estradiol and oral progestin combined treatment seemed to have a slower effect, achieving normalized cycles over subsequent cycles.

COC treatment had a higher patient compliance and satisfaction rate than combined oral progestin and transdermal estrogen treatment, though both treatment groups were considered successful in treating idiopathic AUB-O.

Since our study compared two treatment modalities in a very specific group of patients, the study population was limited. Recall bias should be noted due to the treatment assessment questionnaire being done retrospectively. Because our study assessed patients within a six-month period, lesser common side effects such as venous thromboembolism or malignancies could not be evaluated. A longer follow-up period for a larger patient population is needed for further evaluation.

## Conclusions

COC treatment seems to have better success in adolescent patients when compared to studies on adults; however, there are highly variable results. There is a need for more comprehensive, larger studies on newer treatment modalities for AUB-O in adolescents.

Treatment choice is dependent on many parameters in adolescents with AUB. An immature hypothalamo-pituitary-ovarian axis results in various clinical presentations in adolescence and proves difficult to choose appropriate treatment. Side effects lowering quality of life together with life-threatening side effects should be considered, and patients should be informed on these prior treatment choices.

## Appendices

Supplementary material 1

Patient Questionnaire (Translated From Turkish Original)

1. How long did it take for your menses to become regular with treatment? Please choose one each from two groups of menstrual cycles and months.

o My menses became regular after the 1st menstrual cycle under treatment.

o My menses became regular after the 2nd menstrual cycle under treatment. My menses became regular after the 3rd menstrual cycle under treatment.

o My menses became regular after the 4th menstrual cycle under treatment.

o My menses became regular after the 5th menstrual cycle under treatment.

o My menses became regular after the 6th menstrual cycle under treatment.

o My menses became regular after the 1st month of treatment.

o My menses became regular after the 2nd month of treatment.

o My menses became regular after the 3rd month of treatment.

o My menses became regular after the 4th month of treatment.

o My menses became regular after the 5th month of treatment.

o My menses became regular after the 6th month of treatment.

2. How long did it take for your heavy menstrual bleeding (HMB) to resolve with treatment? Please choose one each from two groups of menstrual cycles and months. (Skip this question if you did not have HMB)

o My HMB resolved after the 1st menstrual cycle under treatment.

o My HMB resolved after the 2nd menstrual cycle under treatment.

o My HMB resolved after the 3rd menstrual cycle under treatment.

o My HMB resolved after the 4th menstrual cycle under treatment.

o My HMB resolved after the 5th menstrual cycle under treatment.

o My HMB resolved after the 6th menstrual cycle under treatment.

o My HMB resolved after the 1st month of treatment.

o My HMB resolved after the 2nd month of treatment.

o My HMB resolved after the 3rd month of treatment.

o My HMB resolved after the 4th month of treatment.

o My HMB resolved after the 5th month of treatment.

o My HMB resolved after the 6th month of treatment.

3. Have you experienced any side effects through the first 3 months of your treatment? If so, select from the options below. More than one option can be selected.

o Headaches

o Abdominal pain

o Nausea-vomiting

o Intermenstrual bleeding

4. Have you experienced any side effects through the second 3 months (4 to 6 months) of your treatment? If so, select from the options below. More than one option can be selected.

o Headaches

o Abdominal pain

o Nausea-vomiting

o Intermenstrual bleeding

5. Have you had any difficulty complying with your treatment?

o Yes

o No

6. Did you at any time of your treatment, have to discontinue the treatment due to side effects?

o Yes

o No

7. Did your presenting symptoms (irregular menstruation/heavy menstrual bleeding/infrequent menstruation) recur within the first 3 months after treatment cessation?

o Yes

o No

8. Did your presenting symptoms (irregular menstruation/heavy menstrual bleeding/infrequent menstruation) recur within the second 3 months (4 to 6 months) after treatment cessation?

o Yes

o No

9. Overall, were you satisfied with your treatment?

o Yes

o No
